# Assessing acoustic competition between sibling frog species using rhythm analysis

**DOI:** 10.1002/ece3.7713

**Published:** 2021-05-27

**Authors:** Alannah Filer, Lara S. Burchardt, Berndt J. van Rensburg

**Affiliations:** ^1^ Centre for Biodiversity and Conservation Science School of Biological Sciences The University of Queensland St Lucia Qld Australia; ^2^ Museum für Naturkunde ‐ Leibniz Institute for Evolution and Biodiversity Science Berlin Germany; ^3^ Animal Behavior Lab Freie Universität Berlin Berlin Germany; ^4^ Department of Zoology University of Johannesburg Johannesburg South Africa

**Keywords:** acoustic competition, call rate, eastern sedgefrog, rhythm, wallum sedgefrog

## Abstract

Male frog advertisement calls are species‐specific vocalizations used to attract females for breeding. However, it is possible for environmental or biological sounds to overlap these calls in both frequency and duration resulting in signal confusion, influencing female decision and/or location abilities. It is therefore important for vocal species competing for the same acoustic space to partition their calls either spatially or temporally (via call alternation or suppression). However, frog species previously isolated from each other may not have developed appropriate adaptive behaviors, resulting in acoustic competition. This study applied rhythm analysis to track changes in calling behavior, namely changes in calling frequency (as in beats per second), of the wallum sedgefrog and the eastern sedgefrog when vocalizing alone versus in the presence of each other to assess potential acoustic competition. Our main findings demonstrated that both species significantly altered their calling behavior when exposed to each other. While we expected the increased calling activity of one species to inhibit the activity of the other to avoid signal confusion, we instead found that both species greatly increased the beat frequency of their calls when calling in the presence of each other. We also found evidence of beat frequency development in the wallum sedgefrog whereby there was always a strong initial increase in call frequency in reaction to the first vocal interruption by the eastern sedgefrog. These results support the hypothesis that the eastern sedgefrog and the wallum sedgefrog are in competition for the acoustic space in habitats where they occur together. This highlights a new threat to the vulnerable wallum sedgefrog species and may serve to inform future management practices. Using rhythm analyses to track changes in acoustic behavior can help inform on important population dynamics such as health, trajectory, and response to management, and therefore be of great benefit to the conservation of vocal species.

## Graphical abstract

Male frog advertisement calls are used to attract females for breeding, and it is therefore imperative that the males are able to signal without interference from overlapping or masking calls produced by other species. We used rhythm analysis to observe and track changes in the calling behavior of two competing frog species when they were calling alone and in a shared habitat. We observed that both species increased the beat frequency of their calls in reaction to the presence of the other frog, indicating a possible response to acoustic competition.

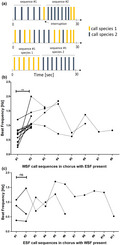

## 1 INTRODUCTION

Frog advertisement calls are loud species‐specific calls produced by males to attract females for breeding purposes (Gerhardt & Huber, 2002; Littlejohn & Martin, 1969; Melendez, 2008; Narins, 2007). Females use these calls to identify and locate male individuals of their own species, particularly in vegetated landscapes (Littlejohn & Martin, 1969; Narins, 2007). Components of these calls such as frequency and/or repetition rate may provide an indication of the male's fecundity (e.g., body size), as well as give an approximation of the chorus density (Narins, 2007). For anurans relying on advertisement calls for breeding, it is vital for the males of the species to be able to occupy at least a portion of the local acoustic space without signal confusion (Narins, 2007). In accordance with this, the acoustic adaptation hypothesis poses that vocal animals adjust the timing or structure of their acoustic signals to limit degradation when broadcasting due to environmental or biological conditions or interference (Ey & Fischer, 2009).

While advertisement calls are species‐specific, it is possible for heterospecific call frequency or call duration overlap to occur between two or more species, which may result in signal confusion, influencing female decision processes or locating abilities (Littlejohn & Martin, 1969; Narins, 2007). Frog species occurring in sympatry therefore often call at differing times in the season or day, or may alternate calls in order to limit signal overlap within the acoustic space (Littlejohn & Martin, 1969; Martínez‐Rivera & Gerhardt, 2008). However, frog species previously occurring in parapatry may not have developed such complex temporal partitioning in their calling behavior, resulting in direct call competition when these species become sympatric. This competition is likely to occur at times of peak calling activity when breeding success is at its highest, that is, following periods of rainfall when ephemeral pools of water are present, or temporally during the night when predator and desiccation risk is reduced (Oseen & Wassersug, 2002; Saenz et al., 2006).

Acoustic competition has been speculated to occur between the sibling species *Litoria olongburensis* and *Litoria fallax*, also known as the wallum sedgefrog and the eastern (common) sedgefrog. The wallum sedgefrog is endemic to coastal eastern Australia and is dependent on the highly specialized wallum wetlands currently under threat by land use changes, and is consequently listed as vulnerable by the IUCN and under federal legislation (Hines et al., 2004). The wallum wetlands often occur adjacent to or within highly populated coastal areas, and are therefore very susceptible to human influence affecting local water quality and hydrology (Gagné & Fahrig, 2010; Ingram & Corben, 1975; Kikkawa et al., 1979). This habitat is characterized by oligotrophic, acidic waters meaning that it is often only inhabited by a very sparse number of amphibian species other than the group of specially adapted “acid frog” species, to which the wallum sedgefrog belongs (Ingram & Corben, 1975; Kikkawa et al., 1979; Lewis & Goldingay, 2005; Meyer et al., 2006).

However, local disturbance has the potential to cause this previously inhospitable habitat to become more accessible to nonwallum frog species through the alteration of the nutrient load or pH (Meyer et al., 2006). In particular, the eastern sedgefrog, a sibling (closely related, morphologically similar but reproductively isolated) species to the wallum sedgefrog, is a notable example of a species that capitalizes on disturbance to colonize new habitats, including that of the wallum wetlands (Meyer et al., 2006). Once introduced, these species initially occur in sympatry, but as anecdotal observations have noted, the wallum sedgefrog is eventually excluded from what is still considered potentially viable habitat (Shuker et al., 2016). It can therefore be inferred that the eastern sedgefrog may be directly competing with the wallum sedgefrog, excluding them from the area. However, the biological mechanisms that account for the suggested competition between these species are still poorly understood.

Acoustic interference can occur when two or more species calling in the same area have similar call characteristics, whereby the call of one may inhibit the calling activity of the other (Páez et al., 1993). To ensure signal recognition, it is important for the calling individual to be heard clearly without overlap with sounds of a similar frequency. Overlapping signaling from individuals of one or more species can degrade the features of the calls and therefore impair various factors of female mate choice including recognition, detection, localization, and discrimination (Tárano & Carballo, 2016). As a result, in several frog species females show a preference for nonoverlapping calls when available (e.g., Bosch & Márquez, 2001; Schwartz et al., 2001). Therefore, it is mutually beneficial for sexually active males to adjust the timing of their calls to avoid overlap, specifically via call alternation (Tárano & Carballo, 2016). For example, in a study on *Dryophytes avivoca* it was found that the females of the species preferred long calls without overlap when offered various two‐call choice experiments (Martínez‐Rivera & Gerhardt, 2008).

Males advertising simultaneously can create deafening choruses, making it difficult for females to locate specific males (i.e., spatial masking; Kelley, 2004). Due to the propensity of noise levels to fluctuate in natural habitats, it is beneficial for vocal animals to be able to quickly adjust their signals in real time to avoid masking effects. These adjustments might include temporal, spatial, or structural shifts (Halfwerk et al., 2015). *Diasporus diastema* males are a good example of a species that actively adjusts the timing of their calls in response to vocal neighbor individuals to maintain an effective synchronous phase relationship of their call bouts (Capshaw et al., 2018). This effect can also be seen in situations of multiple species calling in a single habitat. For example, *Lithobates clamitans* were observed to utilize fine‐scale temporal partitioning to avoid overlapping their calls with *Lithobates catesbeianus* by alternating their calls instead (Herrick et al., 2018).

Additionally, more aggressive tactics may be employed by vocal species to suppress the calls made by competitors. The presence of another sexually active male of the same species has been shown to have an effect on the amount of advertising, up to and including the complete suppression of calls of the *Xenopus laevis* frog despite no physical contact (clasping) occurring. This suppression was observed to release when both frogs were isolated from each other. This vocal suppression was postulated to be beneficial for the dominant individual in conferring a reproductive advantage by increasing the likelihood of attracting a receptive female (Tobias et al., 2004). This effect was also later replicated using playback techniques, proving that auditory cues alone are sufficient in suppressing calling activity (Tobias et al., 2010). In a similar fashion when exposed to heterospecific calls during a playback experiment, the call rates of male *Oophaga pumilio* frogs were actively suppressed, although this was not the case in the time periods around the sound exposure (Wong et al., 2009).

The introduction of noise stimulus, be it anthropogenic, abiotic, or biotic, can also have a direct impact on the calling intensity, or rate, of the subject species. However, the response of each species to interfering noise appears to be species specific with several different responses being observed, ranging from increased or neutral to decrease call rates. For example, in a study of an assemblage of frog species in Thailand, Sun and Narins (2005) found a mixed response to external noise stimuli. When exposed to anthropogenic noise three acoustically active species in the assemblage decreased their calling rate, while another (*Hylarana taipehensis*) called more rapidly in response. *Rana taipehensis* also took advantage of quiet periods in the chorus when other frog species were silent, suggesting that interfering noises can affect a chorus both directly by modulating or inhibiting calls, and indirectly by allowing opportunist species to take advantage of resultant acoustic lulls (Sun & Narins, 2005; also see Bosch & Márquez, 2010; Halfwerk et al., 2015; Kaiser & Hammers, 2009; and Lengagne, 2008 for more examples of rate change in response to acoustic stimuli).

Due to similarities in the frequency range (in terms of pitch) of the calls of the wallum sedgefrog and the eastern sedgefrog, it has been proposed that the mechanism of the possible (or speculated) competition between these two sibling species may arise from direct competition for the acoustic space, limiting the breeding success of the wallum sedgefrog in areas of sympatry with the eastern sedgefrog. They therefore provide an excellent opportunity to examine the ability of acoustic analyses, namely rhythm analysis, to determine and exhibit evidence of potential acoustic competition.

Focusing on species with similar call frequencies (pitch), this study examines the rhythm of signaling activity associated with the presence of a sibling species as a possible response to competition for the acoustic space. In order to determine whether the presence of the sibling species has an effect on the calls of the acid frog species (or vice versa), we employed rhythm analysis to track changes in the call frequencies (calls per second, a parameter we refer to as “beat frequency”) for both species. If, as hypothesized, acoustic competition occurs between these two species, a significant change in the beat frequency of one, or both, species’ calling activity would be expected when exposed to the calls of the other, compared to when calling in isolation. This research will provide important evidence on the response of species to acoustic competition in a collapse of situations of parapatry, causing previously discrete species to compete for acoustic space, among other resources. As anthropogenic related disturbance events will likely increase in coming years due to human population growth and consequent land use changes, the introduction of foreign species to previously inaccessible environments is more likely, and it is therefore important to understand the possible consequences of these situations so that vulnerable calling species, such as the endangered wallum sedgefrog can be properly monitored and protected (Ingram & Corben, 1975; Meyer et al., 2006).

## 2 MATERIALS AND METHODS

### 2.1 Study area

Specialized wallum wetland habitat typically occurs along the coastal sandy lowlands of southeast Queensland extending into northeastern New South Wales, <8 m above sea level (Bryan, 1973; Coaldrake, 1961). In Queensland, this habitat forms a narrow strip, 3–50 km wide, between the shoreline and the foothills of the coastal ranges (Bryan, 1973; Coaldrake, 1961).

Pond sites used in this paper occur on the mainland within this habitat band just north of Bribie Island. They include a pond within the protected Glass House Mountains National Park, three artificially created offset ponds within a development site, and a retained natural pond within the same area. These five ponds encompass varying assemblages of the wallum sedgefrog and eastern sedgefrog, both together and separately, with differing population sizes.

### 2.2 Acoustic recordings

Male wallum sedgefrog calling activity typically peaks immediately following wetland inundation and is therefore highly seasonal and strongly influenced by the timing and amount of rainfall (Griffith et al., 2008; Lowe et al., 2015, 2016). This period typically occurs in the summer between October and March (Lowe et al., 2016). Similar to the wallum sedgefrog, male eastern sedgefrogs are at their most vocal in the summer between September and March, particularly on warm nights following rain (Lemckert et al., 2013).

Acoustic data were obtained based on long‐term acoustic recordings using passive SM3 digital audio recorders (Song Meter SM3, Wildlife Acoustics; using internal built‐in stub microphones, gain = 24 dB, and sample rate = 24 kHz). The recordings were conducted during the 2018–2019 and 2019–2020 breeding seasons (October–May) to coincide with periods of higher rainfall that are typically associated peak calling activity for our target species (Lemckert et al., 2013; Lowe et al., 2016). These recordings were largely 10‐min samples, with a selection of 2‐min sub samples. Recordings were chosen for analysis that contained active calling events, but without excessive activity such that individual calls could not be distinguished.

### 2.3 Acoustic preprocessing

**FIGURE A1:**
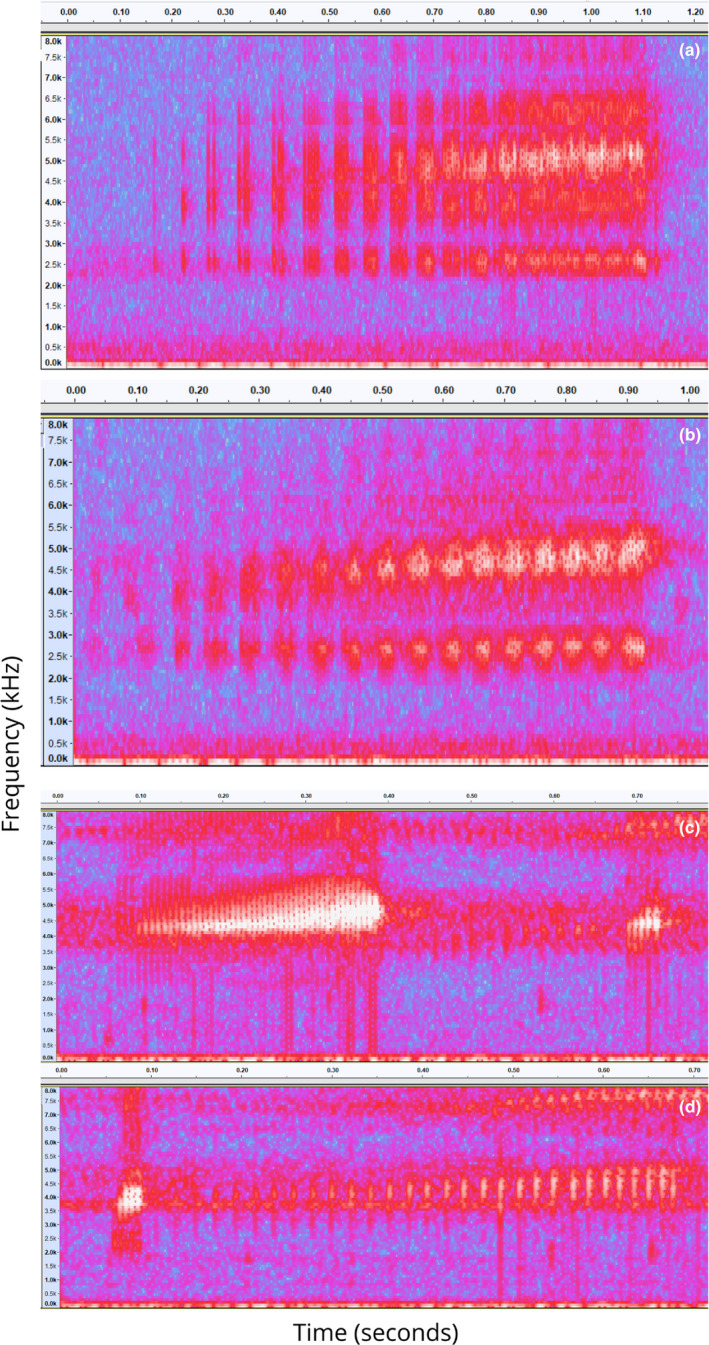
Spectrogram of typical calls of a & b: the wallum sedgefrog (WSF) and c & d: the eastern sedgefrog (ESF). Two examples of each species are shown due to natural variation in call structure. The spectrograms of ESF calls include both call components: the main body and the secondary “click/pip.” Each spectrogram generated in Audacity is shown in color where amplitude (dB) is indicated by an increasing scale of blue to red to white. Frequency (kHz) is shown on the *Y* axis, and time (s) on the *X* axis. Algorithm = Frequencies, Window Size = 256, Window type = Hanning, Zero padding factor = 1

Individual vocalizations of the wallum sedgefrog and the eastern sedgefrog were annotated manually by an expert using Audacity (Audacity Team, 2018). As the eastern sedgefrog has two distinct sections to its call (the main body “riiii i i i i …” and a following “click/pip”; Vanderduys, 2012), only the main body of the call was used in the analysis due to the irregularity in the production of the secondary “click/pip” as well as its very short length (~0.03 s). In contrast, the wallum sedgefrog only has one distinct call body described as a rising, quavering “riiiiiii i i i” (Vanderduys, 2012; see Figure A1 for a spectrogram of both species’ calls). Analyses of the IOI values were performed using the end times of the vocalizations, as this is the most easily delineated point of both species’ calls in the spectrogram, and could therefore be tagged with a much greater confidence (within the nearest 0.01 of a second). The call tags were exported to a csv file where sequences were then identified using a set protocol; sequences had to contain at least five sequential uninterrupted calls from the same species and contain no periods of silence >50 s between calls. Calls in a single sequence are most likely uttered by multiple individuals.

### 2.4 Rhythm analysis

Method instructions were followed as suggested by Burchardt and Knörnschild (2020). IOIs were used as the basis for all analyses but were adjusted to be End‐to‐End Intervals within a sequence, as opposed to the beginning of one call to the beginning of the next, owing to the more definitive structure of the species’ call at this point. A visual inspection of IOI distribution in a histogram was followed by calculations of nPVI values and the coefficient of variation per sequence (for more detailed information on equations on nPVI and coefficient of variation, see Burchardt & Knörnschild, 2020). In this study, we report on two connected parameters related to rhythm analysis: IOIs (of the whole dataset) and exact beat frequencies (in Hertz, beats per second) of individual call sequences; the first parameter being an indicator of the applicability of exact calculations, and the second being the results of those exact calculations. To determine the beat of a particular sequence, the mean IOI of each sequence was converted into the corresponding beat frequency by taking the inverse IOI (i.e., dividing 1 by the mean IOI, as Hertz is 1/s). The development of beats between sequences within one chorus (referred to as “beat development”) was also analyzed.

### 2.5 Statistics

The raw duration of IOIs and the beat frequency of sequences of the two species and different recording situations were compared using an unpaired *t* test with Welch correction (no multiple testing). The beat development within a chorus was assessed using a one‐tailed Wilcoxon signed rank test for the comparison of the first and second sequence (in GraphPad Prism, version 5). Effect sizes were determined in R using the package “effsize” (R version 4.0.1; RStudio version 1.3.959).

## 3 RESULTS

### 3.1 Initial analysis and IOIs

**FIGURE 1:**
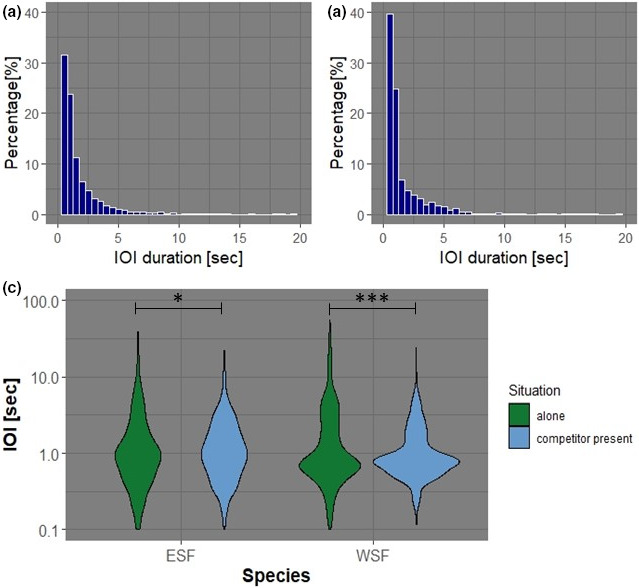
The percentage of IOI durations for (a) the wallum sedgefrog (WSF), and (b) the eastern sedgefrog (ESF). (c) Violin plots of IOI durations (i.e., the time from the end of one call to the end of the next within a sequence) discriminating between situations with both frog species present (competitor present; blue) or only a single frog species present (alone; green) on a logarithmic scale; *p ≤ 0.05, **p ≤ 0.01, ***p ≤ 0.001

The calling activity of both species can be well described with isochronous rhythms. The percentage distribution of IOI durations hints at an underlying isochronous pattern between the two species examined due to the steep unimodal distributions evidencing similar IOIs, the basis for an isochronous beat (Figure 1a,b). The analysis of the nPVI value and coefficient of variation furthermore do not disprove an underlying isochronous beat (see Table A1). Even though individual sequences show high nPVI and coefficient of variation values, most sequences show variability parameters supporting the assumption of an underlying isochronous pattern. It is therefore appropriate to calculate exact beat frequencies for single sequences. When looking at the distribution of IOI durations using violin plots when one frogs species calls on its own, as compared to when it is calling in the presence of the competitor species, longer gaps between calls can be observed when no competitor species is present (Figure 1c; note log‐scale on the y‐axis). The maximum gap between calls for the wallum sedgefrog calling alone is 55.69 s (note that sequences with a silent break longer than 60 s were regarded as two separate sequences); however, when the competitor eastern sedgefrog was present it dropped to 24.21 s. For the eastern sedgefrog, these values were 38.86 s and 22.17 s, respectively. The mean of IOI differs significantly between calls of the wallum sedgefrog when the eastern sedgefrog was present compared to the wallum sedgefrog calling alone. This is also true for the same comparison in the eastern sedgefrog (unpaired *t* test with Welch correction: wallum sedgefrog alone versus. competitor present: *p* < 0.0001***, eastern sedgefrog alone vs. competitor present: *p* = 0.014*). It is suggested that we will therefore also see differences in the exact beat frequencies (in Hz) of individual sequences for the different situations.

### 3.2 Calling dynamics and rhythms

**FIGURE 2:**
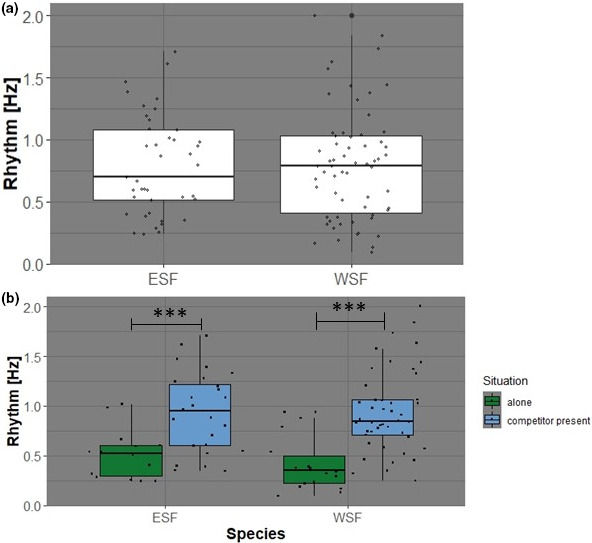
(a) Comparison of call beat (Hz) between all eastern sedgefrog (ESF) and wallum sedgefrog (WSF) calls. (b) Comparison of call beats (Hz) between the ESF and WSF when calling alone (without the presence of a competitor species; green), and when calling in the presence of their competitor species (blue). *p ≤ 0.05, **p ≤ 0.01, ***p ≤ 0.001

**FIGURE A2:**
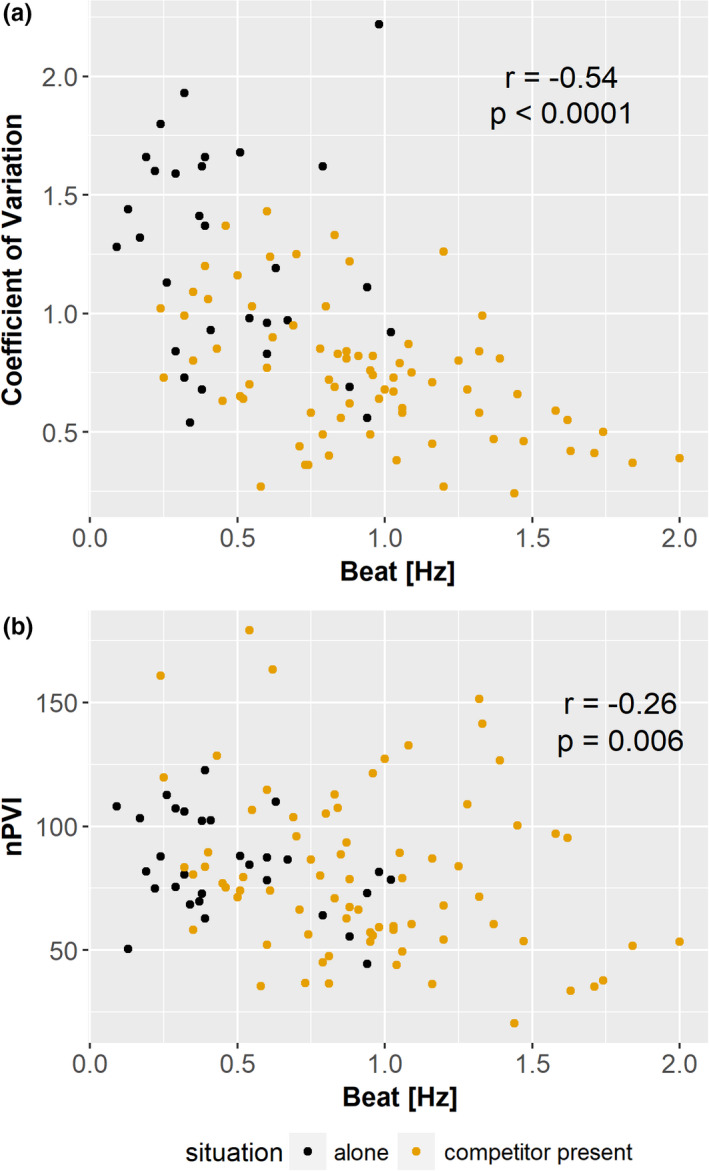
Correlation of variability parameters and beats per analyzed sequence (*n* = 112). The higher the variability, the lower the calculated beats. Shown in black are sequences with only one frog species present (either the eastern sedgefrog or the wallum sedgefrog), and in yellow are sequences from one of the two frog species when in the presence of their respective competitor. (a) Negative correlation of the variability parameter coefficient of variation and calculated beat frequencies in Hertz, *r* = −0.54, *p* > 0.0001***, Pearson correlation. (b) Negative correlation of the variability parameter normalized Pairwise Variability Index (nPVI) and calculated beat frequencies in Hertz per sequence, *r* = −0.26, *p* > 0.006***, Pearson correlation

The analysis of beats based on IOIs provided insight into the dynamics of calling frequency in dependence of the presence of a competitor. Comparing the beat frequency (in Hz) between species, there was no significant difference between the wallum sedgefrog and the eastern sedgefrog (Figure 2a). The beat frequency values range from 0.094 Hz to 2.00 Hz with a mean of 0.80 Hz for the wallum sedgefrog, and from 0.24 Hz to 1.71 Hz with a mean of 0.80 Hz for the eastern sedgefrog. However, if the data are split into instances when each species was calling alone versus in presence of their respective competitor (Figure 2b), a clear difference in the calls' beat frequency is discernible between the situations. For both species, the calling frequency (i.e., the beat frequency) accelerates significantly when the competitor is present and vocally active. These quantitative differences are summarized in Table 1. It should also be noted that higher beat frequencies are correlated with smaller variability parameters (Pearson correlation, nPVI: *r* = −0.26, *p* = 0.006; coefficient of variation: *r* = −0.54, *p* < 0.0001; see Figure A2 for more detail). The higher the beat frequency, the more confident we are in assuming an underlying isochronous pattern.

**TABLE 1:** Beat of the calls of the wallum sedgefrog (WSF) and the eastern sedgefrog (ESF) when calling alone and together

Group	ESF only	ESF when WSF present	WSF only	WSF when ESF present
Minimum	0.24	0.35	0.094	0.24
Maximum	1.02	1.71	0.94	2.00
Mean	0.52	0.94	0.43	0.93
Std	0.25	0.39	0.28	0.42

Note

All beat values presented are in Hz.

There was a significant increase in beat frequency (Hz) in sequences for both species. This increase was stronger in the wallum sedgefrog (unpaired *t* test with Welch correction; wallum sedgefrog alone versus. competitor present: *p* < 0.0001***, *t* = 5.385, *df* = 42; eastern sedgefrog alone vs. competitor present: *p* = 0.0002***, *t* = 4.222, *df* = 36). This was also evidenced in the effect size of the comparison. The difference in situation (alone vs. competitor present) shows a large effect on the beat frequency of calling for both species: *d* = −1.22 for the eastern sedgefrog and *d* = −1.38 for the wallum sedgefrog (Cohen's *d*, 95 percent confidence interval: lower = −1.93, upper = −0.50 and lower = −1.99, upper = −0.77, respectively).

### 3.3 Correlations and beat development within a chorus

Considering these results, it was postulated that there may be a specific parameter driving this increase in beat frequency. However, no clear correlations between the beat of a sequence and parameters including competitor calls in 10 or 20 preceding or subsequent calls, or proportion of competitor and own calls within one recording could be found.

**FIGURE 3:**
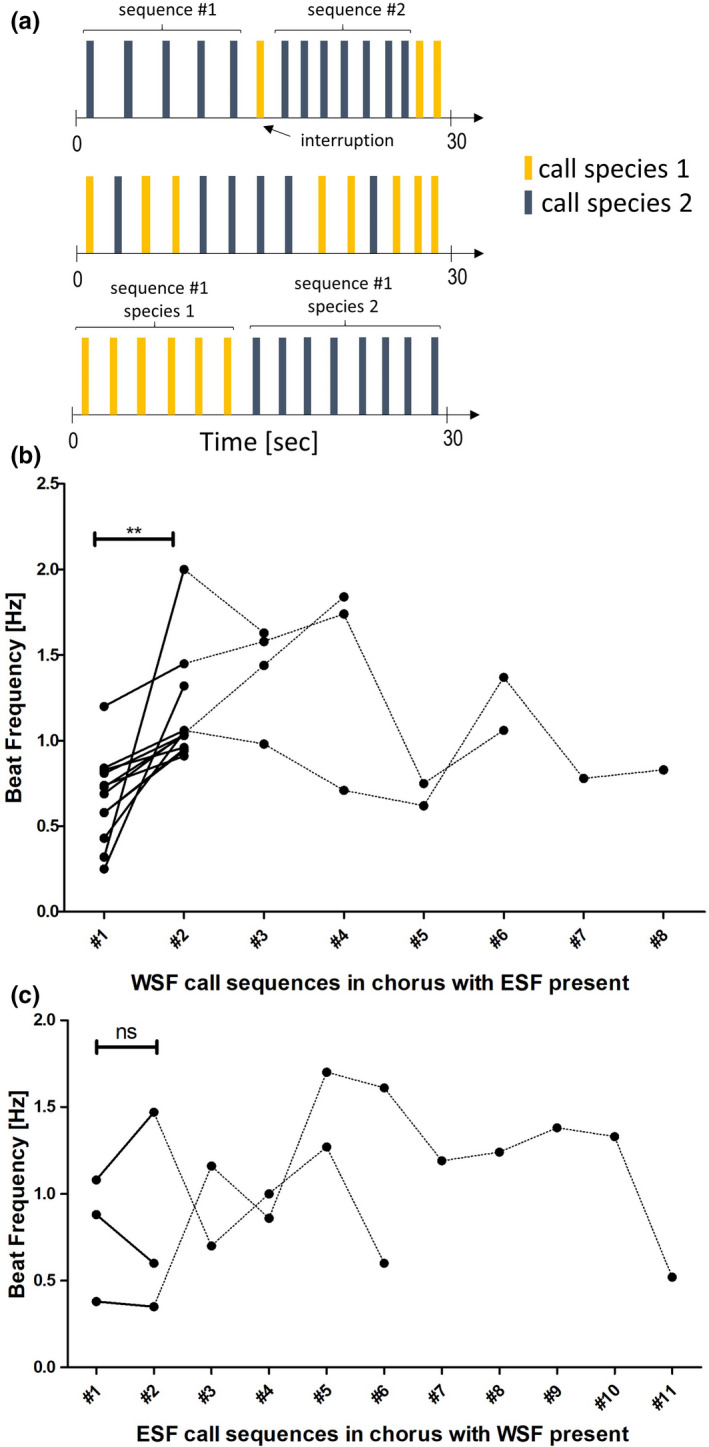
Sequences within a chorus of both frog species. (a) Different scenarios of calling activity within an excerpt of a chorus (calls of the same species are depicted in the same color). This includes: 1. Different sequences of one species being interrupted by the competitor, 2. Alternating calls with too few calls in series to reach the sequence criterion of five subsequent calls, or 3. A sequence of one species being followed by a sequence of the second species. (b) Beat development of wallum sedgefrog (WSF) beat frequencies within a chorus. The beat frequency clearly accelerates from the first to the second sequence (*p* = 0.0013**, Cohen's *d* = −1.67). In subsequent sequences, the trend is not always replicated. (C) Beat development of eastern sedgefrog (ESF) beat frequencies within a chorus. No clear beat development within sequences of this species within one chorus was observed (*p* = 0.5)

Upon further investigation, a more specific beat development within recordings was identified. A typical chorus of both species begins with one species calling and, unless it is interrupted directly (within five calls) by the competitor species, a sequence of continuous calls of one species is created. This is eventually interrupted by a call from the other species, or a break of >60 s (Figure 3a). It was observed that the wallum sedgefrog would typically have a “normal” baseline beat frequency when it was calling early in the chorus, but following an interruption by the eastern sedgefrog (even by a single call), the next sequence of wallum sedgefrog calls increased in tempo dramatically (Figure 3b), this is referred to this as the “beat development within a chorus.” This pattern was evidenced in all examples of the wallum sedgefrog being disrupted after the first sequence by the eastern sedgefrog, leading to a significant increase in tempo (one‐tailed Wilcoxon signed rank test, *p* = 0.0013**, *W* = −78, Table A2). The effect size of this change was considerably large with a value of *d* = −1.67 (Cohen's *d*, 95 percent confidence interval: lower = −3.14 upper = −0.20, Table A2). The escalation was not observed to continue to increase further with more disruptions and subsequent wallum sedgefrog call sequences (Figure 3a), and due to the small sample size, no statistical analysis was run to confirm this visual impression.

Even though the same general reaction of an increase of beat frequency when the competitor was present was observed for the eastern sedgefrog, the same clear beat development between sequences, as seen in the wallum sedgefrog, could not be proven (Figure 3c). There was no statistical difference between the first call sequence and the second call sequence (one‐tailed Wilcoxon signed rank test, *p* = 0.5, *W* = −3, Table A2).

## 4 DISCUSSION

Both the wallum sedgefrog's and the eastern sedgefrog's calls exhibited similar unimodal distributions, evidencing that the call production was in a mostly rhythmic fashion, as the intervals between calls were typically of a similar length (as described by the IOIs in Figure 1a,b). Because of this, the use of the subsequent methods to analyze the calls' beat frequencies were well supported. Additionally, both species were found to share similar beat frequencies (Figure 2a), and the distribution of the IOIs exhibited a strong trend toward longer breaks between calls when each species was calling in the absence of their potential competitor, most significantly in the wallum sedgefrog (Figure 1c).

Acoustic interference occurs when multiple species vocalizing in the same habitat share similar call characteristics, in which the calling activity of one species may affect the activity of another (Páez et al., 1993). As the wallum sedgefrog and the eastern sedgefrog call at a similar frequency (pitch), and have significant overlap in their breeding seasons and calling periods (typically in the evenings after periods of rain/habitat inundation; Lemckert et al., 2013; Lowe et al., 2015, 2016), there is a high potential for acoustic interference and therefore competition. This interference can impair female mate choice including recognition, detection, localization, and discrimination of calls (Tárano & Carballo, 2016). Therefore, changes in the calling behavior of one or both species would be expected, to indicate that a response to acoustic interference was occurring. For example, when exposed to introduce noise and conspecific calls overlapping the species’ call frequency range, male *Engystomops pustulosus* frogs increased call rate, amplitude, and complexity (a response not repeated when exposed to nonmasking noise outside of their frequency range; Halfwerk et al., 2015).

Using rhythm analysis, a very significant change in the calling behavior of both the eastern sedgefrog and the wallum sedgefrog was detected when comparing calls' beat frequencies for both species when in the presence of each other versus alone (Figure 1c, Table A2, Figure 2b). This supports the initial hypothesis that their presence in a shared habitat would result in competition for the acoustic space. Both species were observed to increase the beat frequency of their calls in response to one or more calls of the other species (Figure 1c, Table A2, Figure 2b), although the response was stronger in the wallum sedgefrog than the eastern sedgefrog (Figure 1c, Figure 2b). Increasing call rhythm would be energetically costly for both species as producing advertisement calls is one of the most energetically expensive activities for amphibians (and other ectothermic vertebrates; Anichini et al., 2018; Kruger & Du Preez, 2016). However, as the wallum sedgefrog was shown to increase their beat frequency to a significantly greater extent than the eastern sedgefrog (Figure 2b), it can therefore be inferred that they would consequently confer a higher energetic cost for this adaptation, potentially affecting their fitness as males in poorer condition produce less attractive signals and will not be able to signal for as long as a more efficient caller (Anichini et al., 2018; Kruger & Du Preez, 2016). This in turn may influence their breeding success and long‐term persistence in shared habitats.

In the literature, it can be seen that anuran species can vary in their responses to acoustic stimuli, including the presence of heterospecific calls (see Halfwerk et al., 2015; Kaiser & Hammers, 2009; Lengagne, 2008; Sun & Narins, 2005 for examples of varied responses). However, it was expected that the wallum sedgefrog and the eastern sedgefrog would likely have dissimilar responses when calling in the same environment, in order to avoid call overlap or a deafening chorus; as this would be disadvantageous for both species in terms of female mate detection, choice, and localization (Kelley, 2004; Páez et al., 1993; Tárano & Carballo, 2016). This similar response in both species may be a result of their previous isolation from each other due to the natural exclusion of the eastern sedgefrog from pristine wallum wetland habitat. This habitat is usually characterized by acidic, nutrient poor ephemeral ponds which typically excludes most anuran species not of the specialized “acid frog” group (wallum sedgefrog, wallum rocketfrog, cooloola sedgefrog, and the wallum froglet; Ingram & Corben, 1975; Kikkawa et al., 1979; Lewis & Goldingay, 2005; Meyer et al., 2006).

Consequently, either species may not have developed appropriate adaptive measures to combat the interfering calls of their respective competitor after the introduction of the eastern sedgefrog to areas of wallum due to habitat disturbance via anthropological alteration of the water chemistry. This lack of adaptation may be one of the reasons why, anecdotally, the wallum sedgefrog is not often observed to persist long in ponds where colonization by the eastern sedgefrog has occurred, as they may be more strongly affected by the acoustic interference. This may be exacerbated by the fact that the wallum sedgefrog has been noted to be more selective about when it calls; exhibiting a shorter breeding season with their peak calling activity highly influenced by time since rain, as well as the time of day (Griffith et al., 2008; Lowe et al., 2015, 2016). Conversely, the eastern sedgefrog can be heard to call across a broader time period later into the colder nonbreeding season.

Both species are likely increasing their calls' beat frequency in response to the presence of their competitor in order to ensure a sufficient number of calls are being made in the gaps of the competitor's calls for signal reception and location to occur (i.e., to avoid signal masking or confusion). They may also be attempting to flood the acoustic space to actively suppress the calls of the competitor species, thereby dominating the acoustic space to ensure signal reception without the risk of overlap degrading the features of the call (Tárano & Carballo, 2016; also see Tobias et al., 2004; Wong et al., 2009 for examples of call suppression in anurans). It is possible that both strategies are being employed by either one of the species as an action and response effect, although no evidence of signal suppression was visible in either the wallum sedgefrog or the eastern sedgefrog.

In order to properly observe the effect of this acoustic competition, long‐term studies on population persistence and size (along with acoustic activity) should be performed in ponds where both species occur together (particularly in situations of a recent breakdown in parapatry), in order to determine whether the presence of an actively calling competitor has a negative effect on the breeding success of either species (i.e., is there evidence of heterospecific calls negatively affecting female signal reception/location resulting in flow on effects on breeding success).

The wallum sedgefrog is able to persist in degraded ponds where the pH balance of the usually highly acidic water has been altered. That is, while larvae are usually recorded in waters ranging from pH 3.4–4.5 (Hines & Meyer, 2011), the species is known to breed in wallum habitats with an acidity of pH < 6 (Meyer et al., 2006). However, in degraded ponds where this alteration has facilitated the arrival of the eastern sedgefrog, it is not common for the two species to be observed coexisting over long periods of time (Meyer et al., 2006; Shuker et al., 2016). The wallum sedgefrog may experience greater negative effects in areas of cooccurrence due in part to the fitness cost of increasing their call rhythm (Anichini et al., 2018; Kruger & Du Preez, 2016) and the potential influence of call masking on breeding success (Kelley, 2004), but also due to other compounding mechanisms such as competition for food sources (e.g., small arthropods; Curtis, 2012), perch substrate use (Shuker & Hero, 2012), and potential asymmetrical predation of the wallum sedgefrog and the eastern sedgefrog by other introduced species in disturbed areas such as the eastern mosquitofish (*Gambusia holbrooki*; Meyer et al., 2006; Remon et al., 2016). Therefore, acoustic competition as examined in this study may only be one of many contributing factors to the exclusion of the wallum sedgefrog from still potentially viable habitat.

There has been suggestion of possible wallum sedgefrog hybridization with the Cooloola sedgefrog (*Litoria cooloolensis*), another acid frog that shares a similar morphology, perch use, breeding season, and habitat preference, due to observations of amplexus activity between the species (although no subsequent egg deposition was noted to occur; Lowe & Hero, 2011). These two species, along with the eastern sedgefrog and the northern dwarf treefrog (*Litoria bicolor*), form a group of highly morphologically similar and genetically related tree frogs (Duellman et al., 2016; Tyler & Knight, 2020). It can therefore be considered that hybridization between the eastern sedgefrog and the wallum sedgefrog may be possible in areas of shared habitat (due to similarities in morphology and breeding season; Lemckert et al., 2013; Lowe et al., 2016; Tyler & Knight, 2020), although there have been no recorded observations of this occurring to date. Indeed, Loftus‐Hills and Littlejohn (1971) found that a pair of sympatric species differentiated between heterospecific calls using variations in call structure (such as dominant frequency and pulse rate) could be fooled using synthetic calls mirroring homospecific call pulse repetition rate. As our species have no significant differences in call beat frequencies (Figure 2a), and exhibit overlapping frequency bands (in terms of pitch), there might be the possibility of mate confusion and therefore hybridization between the eastern sedgefrog and the wallum sedgefrog.

Hybrids are often a threat to rare and geographically restricted species (such as the wallum sedgefrog) especially in cases where the other parent species are more widespread and abundant (such as the more generalist eastern sedgefrog), due to differences in overall energy costs of wasted reproductive effort (as a product of differing population sizes, and the presence of nearby source populations; Rhymer & Simberloff, 1996). Hybridization can, in turn, cause genetic extinction and inbreeding depression in remaining nonhybrid populations, reducing fitness (Rhymer & Simberloff, 1996). Hybridization itself may also be unidirectional, in that the males of one species may breed with the females of the other, but not vice versa (Rhymer & Simberloff, 1996). For example, it may be possible that female wallum sedgefrogs may breed with male eastern sedgefrogs either producing hybrid, or even nonviable offspring, while female eastern sedgefrogs still mate productively with conspecific males. Fertile hybrids may also only mate (backcross) with one parental species, influencing the genetic pool of the habitat (Rhymer & Simberloff, 1996). Therefore, if present, hybridization between the eastern sedgefrog and the wallum sedgefrog in areas of cooccurrence may have significant effects on the genetic diversity within the habitat, but also in the presence of one or both of the parental species, and could possibly be a contributing cause of local extinctions of the wallum sedgefrog. However, due to similar morphologies (Tyler & Knight, 2020) and call structures (e.g., call beats in Figure 2a), identifying hybrids between these two species would be difficult without genetic testing and would require significant additional research to verify.

An interesting response also observed in this study was the evidence of beat development (i.e., change in call beat frequency along successive sequences of the same recording) within call sequences of the wallum sedgefrog, whereby the time between calls would decrease after exposure to the calls of the eastern sedgefrog (Figure 3b). This is most likely the observable onset of the behavioral response of the wallum sedgefrog to the presence of the eastern sedgefrog, although no progression/escalation in the beat development was found after the initial event. Notably, there was no similarly observable pattern in the eastern sedgefrog sequences in response to calls of the wallum sedgefrog, although this may be explained by the small sample size available, and the slightly weaker behavioral response of the eastern sedgefrog exhibited in Figure 2b. There is potential to increase this sample size in a more controlled environment by using call playback experiments to introduce the calls of the competitor species multiple times to a wallum sedgefrog or eastern sedgefrog chorus and record behavioral responses over time. This experiment could be extended to explore differences between choruses of each species that occur in ponds with and ponds without their competitor species already present, to explore any differences in learned adaptive behavior.

In this study, we were able to show that the presence of the respective competitor frog species had a significant positive effect on the calling rate of both the eastern sedgefrog and the vulnerable wallum sedgefrog. This indicates that both species may be exhibiting a behavioral response to acoustic competition, whereby both species appear to be attempting to dominate the acoustic space, limiting the opportunity of the other species to call while ensuring signal reception of their own calls. However, as both species exhibited the same behavioral response of increasing their call rate (beat frequency), this in turn increases the likelihood of call overlap and a deafening chorus (making it difficult for females to locate calling males, i.e., spatial masking; Kelley, 2004). This may result in negative effects on the ability of both species to successfully send and receive advertisement signals, which may in turn have flow on effects on breeding success. Follow‐up experiments and monitoring of breeding success in ponds with and without the presence of the competitor species would prove beneficial in monitoring these effects and informing on future management, particularly in regards to the wallum sedgefrog, which is listed as a vulnerable species in need of conservation in state and national legislation in Australia.

Using clear and comparable workflows, incorporating multiple variability parameters, and calculating the exact beats describing a sequence of calls to best observe and track changes in calling behavior is a relatively new approach (Burchardt & Knörnschild, 2020; Ravignani & Norton, 2017). This study is novel in its application of this approach on anuran species, and it is the first time that this method has been used to visualize and quantify behavioral responses to acoustic competition. Our main findings not only quantified an additional potential threat related to acoustic competition faced by an already endangered wallum sedgefrog, but also more broadly demonstrates the application value of these rhythm analyses to better understand changes in acoustic relationships and calling behavior between vocal species that are likely to influence critical life history events like mating and recruiting. Monitoring such events can benefit conservation if they provide new insights into a population's state, trajectory or response to management. Our study therefore supports the broader notion that conservation‐relevant information can be derived from acoustic signatures associated with particular behavior (see the concept of “acoustic conservation behavior” in Teixeira et al., 2019).

## CONFLICT OF INTEREST

The authors declare no conflict of interest.

## AUTHOR CONTRIBUTIONS

**Alannah Filer:** Conceptualization (equal); Formal analysis (supporting); Investigation (lead); Writing‐original draft (lead); Writing‐review & editing (equal). **Lara S. Burchardt:** Conceptualization (equal); Formal analysis (lead); Methodology (lead); Visualization (lead); Writing‐original draft (supporting); Writing‐review & editing (equal). **Berndt J. van Rensburg:** Conceptualization (equal); Supervision (lead); Writing‐review & editing (equal).

## Data Availability

The raw sequence and IOI data are available at the open access University of Queensland UQ eSpace data repository at "https://doi.org/10.14264/6c4553a" (https://doi.org/10.14264/6c4553a) and will be available on request from the corresponding author at alannah.filer@uq.net.au.

## 1

**TABLE A1:** Analysis of wallum sedgefrog (WSF) and eastern sedgefrog (ESF) call sequences within each recording

Recording	Sequence	#elements	% WSF calls	% ESF calls	nPVI	Coefficient of variation	Rhythm [Hz]	Situation
1	WSF 1	85	100	0	74.80	1.60	0.22	Alone
	WSF 2	23			103.27	1.32	0.17	Alone
2	WSF 1	16	100	0	102.30	0.68	0.38	Alone
	WSF 2	110			64.06	1.62	0.79	Alone
	WSF 3	19			50.53	1.44	0.13	Alone
3	WSF 1	38	100	0	72.74	1.62	0.38	Alone
	WSF 2	22			108.03	1.28	0.09	Alone
4	WSF 1	481	100	0	73.10	1.11	0.94	Alone
5	WSF 1	108	100	0	81.83	1.66	0.19	Alone
6	WSF 1	190	100	0	66.05	1.35	0.54	Alone
7	WSF 1	14	100	0	107.30	0.84	0.29	Alone
8	WSF 1	38	100	0	80.50	1.93	0.32	Alone
9	WSF 1	21	100	0	69.65	1.41	0.37	Alone
10	WSF 1	23	100	0	62.74	1.66	0.39	Alone
11	WSF 1	51	100	0	55.53	0.69	0.88	Alone
12	WSF 1	55	100	0	44.48	0.56	0.94	Alone
13	WSF 1	18	100	0	68.36	0.54	0.34	Alone
14	ESF 1	83	0	100	87.37	0.96	0.60	Alone
	ESF 2	137			78.47	0.92	1.02	Alone
	ESF 3	206			81.54	2.22	0.98	Alone
15	ESF 1	12	0	100	122.69	1.37	0.39	Alone
	ESF 2	30			109.98	1.19	0.63	Alone
	ESF 3	103			87.79	1.80	0.24	Alone
16	ESF 1	135	0	100	88.04	1.68	0.51	Alone
	ESF 2	25			84.56	0.98	0.54	Alone
17	ESF 1	61	0	100	86.66	0.97	0.67	Alone
	ESF 2	25			78.31	0.83	0.60	Alone
18	ESF 1	23	0	100	102.45	0.93	0.41	Alone
19	ESF 1	14	0	100	75.58	1.59	0.29	Alone
20	ESF 1	15	0	100	112.72	1.13	0.26	Alone
21	ESF 1	10	0	100	106.05	0.73	0.32	Alone
22		98	68.37	31.63				
	WSF 1	9			35.37	0.27	0.58	Competitor present
	WSF 2	48			57.07	0.76	0.95	Competitor present
	ESF 1	14			53.30	0.49	0.95	Competitor present
23		425	5.41	94.59				
	ESF 1	44			60.48	0.75	1.09	Competitor present
	ESF 2	7			53.60	0.46	1.47	Competitor present
	ESF 3	7			95.85	1.25	0.70	Competitor present
	ESF 4	11			127.31	0.68	1.00	Competitor present
	ESF 5	21			108.90	0.68	1.28	Competitor present
	ESF 6	283			73.99	1.24	0.61	Competitor present
24		432	59.26	40.74				
	ESF 1	43			83.57	1.20	0.39	Competitor present
	WSF 1	14			83.43	0.99	0.32	Competitor present
	ESF 2	19			80.50	0.80	0.35	Competitor present
	ESF 3	6			87.02	0.71	1.16	Competitor present
	ESF 4	6			93.42	0.84	0.87	Competitor present
	ESF 5	8			35.27	0.41	1.71	Competitor present
	ESF 6	5			95.42	0.55	1.62	Competitor present
	ESF 7	6			54.20	0.27	1.20	Competitor present
	ESF 8	18			83.76	0.80	1.25	Competitor present
	ESF 9	6			126.53	0.81	1.39	Competitor present
	ESF 10	6			141.31	0.99	1.33	Competitor present
	WSF 2	8			53.34	0.39	2.00	Competitor present
	WSF 3	27			33.57	0.42	1.63	Competitor present
	ESF 11	9			79.42	0.64	0.52	Competitor present
	WSF 4	14			76.87	0.63	0.45	Competitor present
25		190	91.58	8.42				
	WSF 1	66			52.19	1.43	0.60	Competitor present
	WSF 2	6			58.18	1.09	0.35	Competitor present
	ESF 1	9			105.18	1.03	0.80	Competitor present
	WSF 3	90			75.38	1.37	0.46	Competitor present
26		423	5.20	94.80				
	ESF 1	44			60.48	0.75	1.09	Competitor present
	ESF 2	7			53.60	0.46	1.47	Competitor present
	ESF 3	7			95.85	1.25	0.70	Competitor present
	ESF 4	11			127.31	0.68	1.00	Competitor present
	ESF 5	5			179.08	0.70	0.54	Competitor present
	ESF 6	11			151.49	0.84	1.32	Competitor present
	ESF 7	9			36.33	0.45	1.16	Competitor present
	ESF 8	115			78.70	1.22	0.88	Competitor present
	ESF 9	167			71.39	1.16	0.50	Competitor present
27		18	66.67	33.33				
	ESF 1	6			74.12	0.65	0.51	Competitor present
	WSF 1	12						Competitor present
28		35	77.14	22.86				
	WSF 1	8			119.65	0.73	0.25	Competitor present
	WSF 2	6			71.51	0.58	1.32	Competitor present
29		36	19.44	80.56				
	ESF 1	20			106.5	1.03	0.55	Competitor present
30		12	58.33	41.67				
	WSF 1	7			36.58	0.72	0.81	Competitor present
31		97	68.04	31.96				
	WSF 1	9			35.37	0.27	0.58	Competitor present
	WSF 2	48			57.07	0.76	0.95	Competitor present
	ESF 1	14			53.3	0.49	0.95	Competitor present
32		109	76.15	23.85				
	WSF 1	11			36.75	0.36	0.73	Competitor present
	WSF 2	57			58.16	0.73	1.03	Competitor present
	ESF 1	6			132.58	0.87	1.08	Competitor present
33		109	77.98	22.02				
	WSF 1	12			47.57	0.4	0.81	Competitor present
	WSF 2	57			59.68	0.67	1.03	Competitor present
34		87	79.31	20.69				
	WSF 1	11			56.23	0.36	0.74	Competitor present
	WSF 2	46			66.38	0.82	0.91	Competitor present
35		96	67.71	32.29				
	WSF 1	44			62.7	0.81	0.87	Competitor present
	ESF 1	5			121.3	0.82	0.96	Competitor present
36		94	64.89	35.11				
	WSF 1	12			70.84	0.69	0.83	Competitor present
	WSF 2	26			55.91	0.74	0.96	Competitor present
37		54	48.15	51.85				
	ESF 1	17			89.4	1.06	0.4	Competitor present
	WSF 1	7			45.1	0.49	0.79	Competitor present
38		99	68.69	31.31				
	WSF 1	11			103.6	0.95	0.69	Competitor present
	WSF 2	7			44	0.38	1.04	Competitor present
	WSF 3	12			20.55	0.24	1.44	Competitor present
	WSF 4	9			51.73	0.37	1.84	Competitor present
39		58	65.52	34.48				
	WSF 1	6			128.41	0.85	0.43	Competitor present
	WSF 2	18			89.3	0.79	1.05	Competitor present
	ESF 1	8			67.44	0.62	0.88	Competitor present
	ESF 2	6			114.8	0.77	0.6	Competitor present
	WSF 3	8			88.55	0.56	0.85	Competitor present
40		141	78.72	21.28				
	WSF 1	9			68.03	1.26	1.2	Competitor present
	WSF 2	14			100.4	0.66	1.45	Competitor present
	WSF 3	6			97.04	0.59	1.58	Competitor present
	WSF 4	18			37.84	0.5	1.74	Competitor present
	WSF 5	9			86.46	0.58	0.75	Competitor present
	WSF 6	17			49.53	0.58	1.06	Competitor present
41		119	85.71	14.29				
	WSF 1	8			107.4	0.83	0.84	Competitor present
	WSF 2	9			79.1	0.6	1.06	Competitor present
	WSF 3	9			59.2	0.64	0.98	Competitor present
	WSF 4	7			66.3	0.44	0.71	Competitor present
	WSF 5	6			163.2	0.9	0.62	Competitor present
	WSF 6	16			60.4	0.47	1.37	Competitor present
	WSF 7	10			80.1	0.85	0.78	Competitor present
	WSF 8	8			112.8	1.33	0.83	Competitor present
42		23	56.52	43.48				
	WSF 1	6			160.9	1.02	0.24	Competitor present

Note

The number and proportion of calls of each species is reported, along with the nPVI value, coefficient of variation, and rhythm value. Situation is indicative of whether the species was calling alone or in the presence of the competitor species.

**TABLE A2:** Beat development in the rhythm (Hz) of wallum sedgefrog (WSF) and eastern sedgefrog (ESF) call sequences within recordings containing calls of both species

Recoding	Species	#1	#2	#3	#4	#5	#6	#7	#8	#9	#10	#11
27	WSF	0.25	1.32									
30	WSF	0.58	0.95									
31	WSF	0.73	1.03									
32	WSF	0.81	1.03									
33	WSF	0.74	0.91									
35	WSF	0.83	0.96									
37	WSF	0.69	1.04	1.44	1.84							
38	WSF	0.43	1.05									
39	WSF	1.2	1.45	1.58	1.74	0.75	1.06					
40	WSF	0.84	1.06	0.98	0.71	0.62	1.37	1.38	0.78	0.83		
21	WSF	0.58	0.94									
23	WSF	0.32	2	1.63								
												
22	ESF	1.08	1.47	0.7	1	1.27	0.6					
23	ESF	0.38	0.35	1.16	0.86	1.7	1.61	1.19	1.24	1.38	1.33	0.52
38	ESF	0.88	0.6									
